# Annual trends in arthroscopic meniscus surgery: Analysis of a national database in Japan

**DOI:** 10.1371/journal.pone.0194854

**Published:** 2018-04-03

**Authors:** Manabu Kawata, Yusuke Sasabuchi, Shuji Taketomi, Hiroshi Inui, Hiroki Matsui, Kiyohide Fushimi, Hirotaka Chikuda, Hideo Yasunaga, Sakae Tanaka

**Affiliations:** 1 Department of Orthopaedic Surgery, Faculty of Medicine, The University of Tokyo, Bunkyo-ku, Tokyo, Japan; 2 Department of Clinical Epidemiology and Health Economics, School of Public Health, The University of Tokyo, Bunkyo-ku, Tokyo, Japan; 3 Department of Health Informatics and Policy, Graduate School of Medicine, Tokyo Medical and Dental University, Bunkyo-ku, Tokyo, Japan; 4 Department of Orthopaedic Surgery, Gunma University Graduate School of Medicine, Maebashi, Gunma, Japan; Augusta University, UNITED STATES

## Abstract

**Background:**

The importance of meniscus preservation is widely recognized. There have been a few studies describing trends in meniscectomy and meniscus repair in the United States; however, they presented differing results. We reported annual trends in meniscus surgery, using a national database in Japan.

**Methods:**

We interrogated the Diagnosis Procedure Combination database, which represents approximately half of all hospital admissions in Japan. We included the patients who underwent meniscectomy and meniscus repair between July 2007 and March 2015. The diagnosis, age and sex of each patient were recorded.

**Results:**

We identified 83,105 patients: 69,310 underwent meniscectomy; 13,416 underwent meniscus repair and 379 underwent both in a single admission. The proportion of patients undergoing meniscus repair rose from 7.0% in 2007 to 25.9% in 2014 (p < 0.001), while the proportion undergoing meniscectomy fell from 92.8% in 2007 to 73.3% in 2014 (p < 0.001). Among patients under 30 years old, the proportions undergoing meniscus repair or meniscectomy in 2014 were 50.3% *versus* 48.3%, respectively. A bimodal age distribution was observed for meniscectomy, with peaks at 10–19 years of age and 60–69 years of age, whereas most patients undergoing meniscus repair were 10–19 years of age.

**Conclusions:**

We found characteristic trends where the popularity of meniscus repair increased rapidly at the expense of meniscectomy in Japan during the study period. In the last survey year, the proportion of meniscus repair exceeded that of meniscectomy in those younger than 30 years. Meniscectomy was undertaken most often in adolescents and early old age, while meniscus repair was undertaken most often in adolescents.

## Introduction

Knee arthroscopy for meniscus injury is one of the most commonly performed procedures in orthopedic surgery [1,2]. Meniscectomy was the most common arthroscopic procedure undertaken to address the symptoms of a meniscus tear [1,3]. There is the widely accepted recognition that the meniscus is critical to effective load transmission [4,5] and shock absorption [6], and a number of studies have reported that meniscus resection significantly increases the risk of subsequent osteoarthritis [7–11]. Some studies have demonstrated that the clinical outcomes and the long-term cost-effectiveness after meniscus repair are superior to those of meniscectomy [12–15], although it appears to have a higher reoperation rate than partial meniscectomy [11]. Recently, a greater emphasis has been placed on meniscus preservation for the prevention of osteoarthritis, especially in young patients [16–18]. Advances in arthroscopic devices and techniques [14,19,20] have made suturing of the meniscus less technically demanding, allowing repair of a variety of injuries including root tears [21] and horizontal tears [22]. In contrast, partial meniscectomy is generally still performed for the treatment of tears in middle or older age, as age-related degenerative tears are not expected to heal by suturing [23]. Nevertheless, numerous randomized clinical trials have shown that partial meniscectomy has no advantage over non-surgical treatments for middle-aged and older patients [24–29]. Athletes with meniscus tears sometimes undergo partial meniscectomy when they are eager to return to competition quickly and with a lower risk of reoperation [11,30].

Nationwide studies of trends in meniscectomy and meniscus repair illuminate how the clinical evidence base influences the selection of surgical techniques. Three previous studies from the United States showed divergent trends in arthroscopic meniscus surgery; one of them reported a decrease in cases of meniscectomy per surgeons [31], while the other two showed that the incidence of meniscectomy was stable or increasing [16,32]. As for meniscus repair, two of them reported an increase in meniscus repair [16,31], but the proportion undergoing meniscus repair was still substantially lower than meniscectomy, even among young patients [16]. These results indicate that the trends of meniscus surgeries may not fully reflect recent evidence on meniscus treatment. However, these previous studies are mostly from the United States, and may not be generalizable to other countries. Further investigation on this issue is required using data from other countries; also, the previous studies lacked detailed information on patient characteristics undergoing arthroscopic meniscus surgeries [16,31–33].

The objectives of this study were to use a national database to examine recent trends in arthroscopic meniscus surgery in Japan, and establish the characteristics of patients undergoing meniscus surgery. We hypothesized that there would be an increase in the proportion undergoing meniscus repair and a decrease in meniscectomy, especially in young patients.

## Materials and methods

Inpatient data were obtained from the Japanese Diagnosis Procedure Combination (DPC) database. All 82 academic hospitals in Japan are mandatory contributors, but participation by community hospitals is voluntary. The DPC database contains discharge abstract and administrative reimbursement claims data for inpatient episodes collected from participating hospitals, the details of which have been documented elsewhere [34–36]. Data were collected for 6 months (July to December) of each year from 2007 to 2010, but have been collected continuously since 2011. The database conforms to the Japanese academic year, which starts in April and ends in March. Data were therefore entered to the DPC for the following periods: July to December 2007; July to December 2008; July to December 2009; July 2010 to March 2011; April 2011 to March 2012; April 2012 to March 2013; April 2013 to March 2014; and April 2014 to March 2015. In 2014, 1,133 hospitals participated and provided data for 7.82 million patients, representing 56.4% of all inpatient admissions to acute care hospitals in Japan. To optimize the accuracy of the data, doctors in charge are required to document the diagnoses using ICD-10 codes, and the types of surgery or interventions coded by the Japanese original K-code, with reference to the medical records. The Institutional Review Board at our institution approved the study design and waived the requirement for informed consent as all data are anonymous.

All inpatients undergoing arthroscopic meniscectomy (K-code: K068-2), arthroscopic meniscus repair (K-code: K069-3) or both during a single admission were included in our analysis. The following data were extracted from the database: diagnoses, surgical procedures or other interventions during hospitalization, and demographic data such as age and sex.

We first examined annual trends in meniscectomy and meniscus repair during the study period, using data from July 2007 to March 2015, a total of 75 months. Next, we analyzed the data having divided patients into one of three age categories: < 30 years old; 30–59 years old; ≥ 60 years old. Then, we analyzed trends categorized by site of injury: medial; lateral; bicompartmental (those in whom injured compartments were not recorded were excluded from this subgroup analysis). Further, we examined the age distribution of patients undergoing meniscus surgery, taking into account procedures, injured compartments and sex; patients were categorized by their decade of age.

The SPSS statistical software package (version 23.0; IBM, Armonk, NY, USA) and R version 3.1.3 (The R Foundation, Vienna, Austria) were used for all analyses. The Cochran–Armitage test was undertaken for trend analysis. The threshold for significance was < 0.05.

## Results

We identified 83,105 patients who had undergone arthroscopic meniscus surgery during the study period: 69,310 underwent meniscectomy, 13,416 meniscus repair and 379 underwent both during a single admission. Overall, 31,712 patients had medial tears, 20,299 had lateral tears and 399 had bicompartmental tears; the site of injury was not recorded in 30,695 cases.

**Fig 1:**
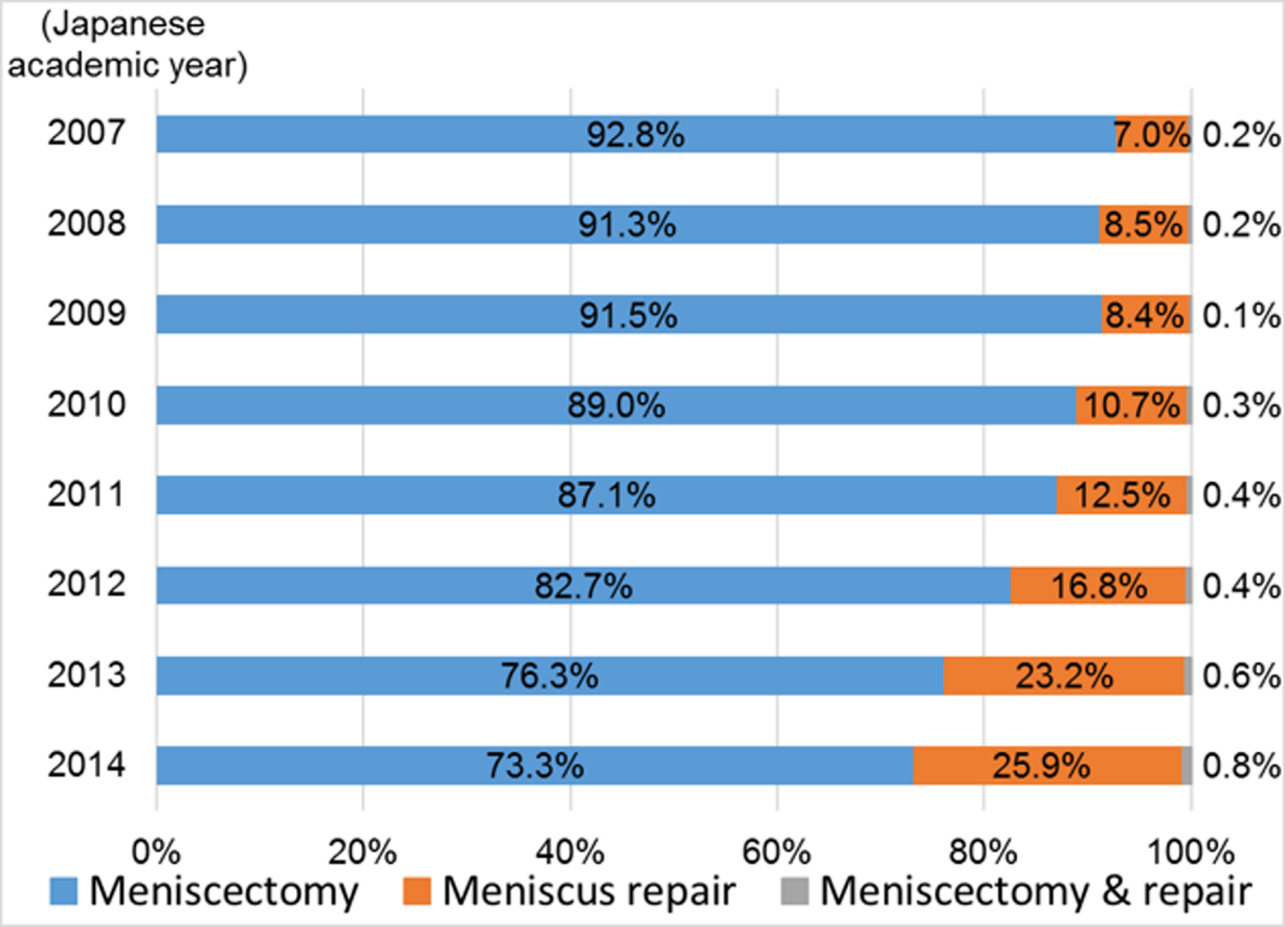
Annual trends in the proportion of meniscectomy and meniscus repair from 2007 to 2014. The Japanese academic year begins April 1 and ends March 31.

**Fig 2:**
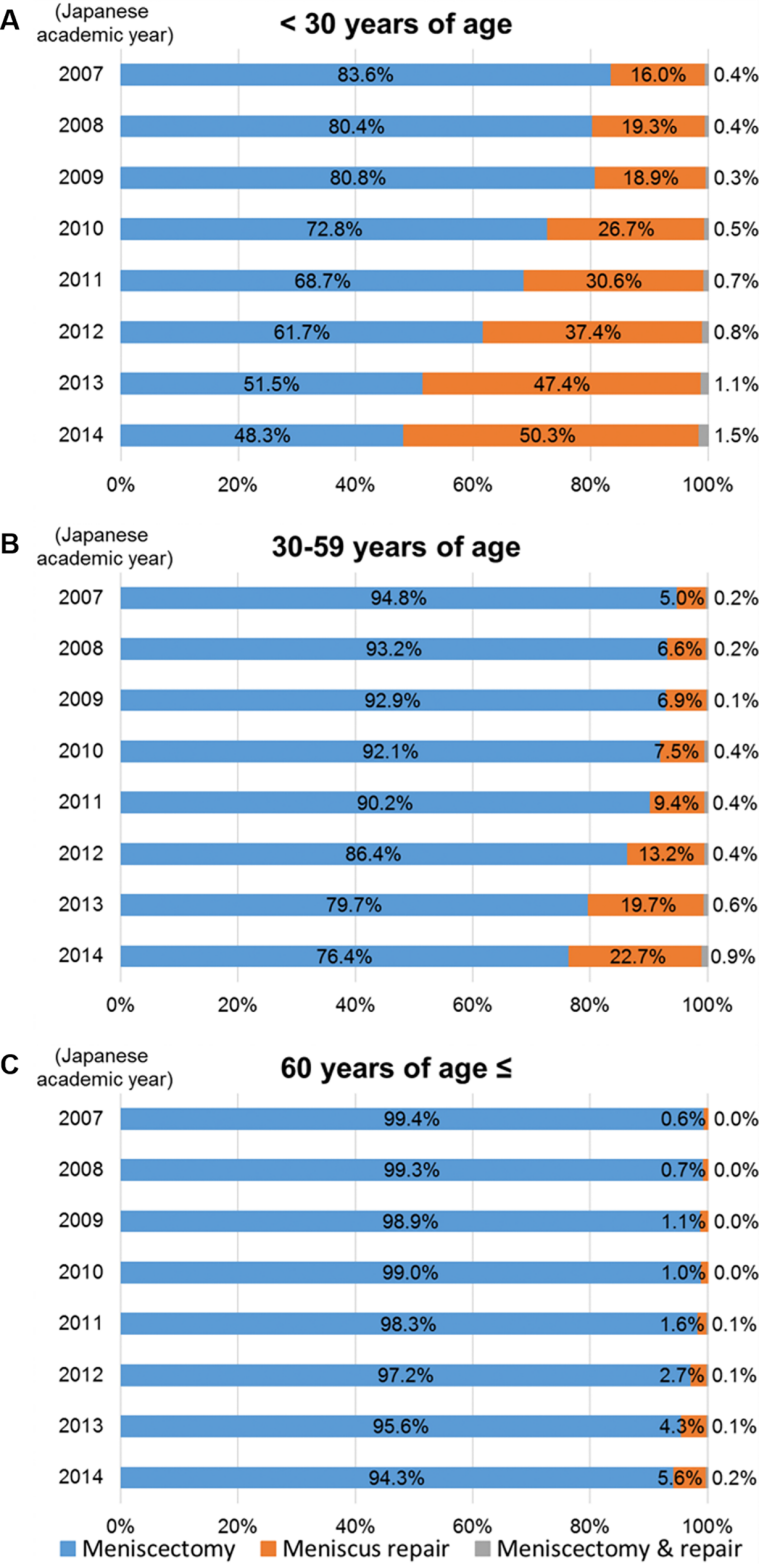
Annual trends in the proportion of meniscectomy and meniscus repair categorized by age. The Japanese academic year begins April 1 and ends March 31. (A) < 30 years old. (B) 30–59 years old. (C) ≥ 60 years old.

There was a more than threefold increase in the proportion of patients undergoing meniscus repair during the study period, rising from 7.0% in 2007 to 25.9% in 2014 (p < 0.001). In contrast, the proportion undergoing meniscectomy decreased from 92.8% to 73.3% during the same period (p < 0.001; Table 1 and Fig 1). The proportions undergoing meniscus repair increased significantly in all age groups (p < 0.001 for all analyses, Table 2 and Fig 2). By 2014, the proportion of patients < 30 years old undergoing meniscus repair increased to exceeding the proportion undergoing meniscectomy (50.3% compared with 48.3%, respectively). Annual trends categorized by injured compartment are shown in Table 3. The proportions undergoing meniscus repair showed significant increases in both medial and lateral tear (p < 0.001 for both analyses), although those of lateral meniscus were larger than those of medial one throughout the study period.

**Table 1:** Annual trends in meniscectomy and meniscus repair from 2007 to 2014.

Japanese academic year^a^		2007	2008	2009	2010	2011	2012	2013	2014	Total
Survey periods (months)		6^b^	6^b^	6^b^	9^c^	12^d^	12^d^	12^d^	12^d^	75
Total patients (millions)^e^		2.7	2.8	2.8	5.0	7.1	6.9	7.1	7.8	42.1
Meniscectomy	Cases	5,997	6,045	5,857	7,054	11,200	11,903	10,651	10,603	69,310
	(%)^f^	(92.8)	(91.3)	(91.5)	(89.0)	(87.1)	(82.7)	(76.3)	(73.3)	(83.4)
Meniscus repair	Cases	450	565	538	847	1,610	2,425	3,234	3,747	13,416
	(%)^f^	(7.0)	(8.5)	(8.4)	(10.7)	(12.5)	(16.8)	(23.2)	(25.9)	(16.1)
Meniscectomy & repair	Cases	13	14	9	25	49	64	83	122	379
	(%)^f^	(0.2)	(0.2)	(0.1)	(0.3)	(0.4)	(0.4)	(0.6)	(0.8)	(0.5)
Total		6,460	6,624	6,404	7,926	12,859	14,392	13,968	14,472	83,105

^a^ the Japanese academic year begins April 1 and ends March 31

^b^ July to December

^c^ July to March

^d^ April to March

^e^ number of patients discharged from all hospitals contributing to the Diagnosis Procedure Combination database during the data collection period for that year

^f^ proportion of all meniscus surgery during the data collection period.

**Table 2:** Annual trends in meniscectomy and meniscus repair categorized by age.

	Japanese academic year^a^		2007	2008	2009	2010	2011	2012	2013	2014	Total
Age, y	Survey periods (months)		6^b^	6^b^	6^b^	9^c^	12^d^	12^d^	12^d^	12^d^	75
< 30	Meniscectomy	Cases	1,526	1,503	1,380	1,548	2,312	2,493	2,094	2,087	14,943
		(%)^e^	(83.6)	(80.4)	(80.8)	(72.8)	(68.7)	(61.7)	(51.5)	(48.3)	(64.1)
	Meniscus repair	Cases	293	360	323	568	1,030	1,512	1,930	2,174	8,190
		(%)^e^	(16.0)	(19.3)	(18.9)	(26.7)	(30.6)	(37.4)	(47.4)	(50.3)	(35.1)
	Meniscectomy & repair	Cases	7	7	5	11	22	34	45	63	194
		(%)^e^	(0.4)	(0.4)	(0.3)	(0.5)	(0.7)	(0.8)	(1.1)	(1.5)	(0.8)
	Total		1,826	1,870	1,708	2,127	3,364	4,039	4,069	4,324	23,327
30–59	Meniscectomy	Cases	2,815	2,742	2,607	3,116	4,996	5,212	4,559	4,494	30,541
		(%)^e^	(94.8)	(93.2)	(92.9)	(92.1)	(90.2)	(86.4)	(79.7)	(76.4)	(86.6)
	Meniscus repair	Cases	147	193	194	255	518	798	1,125	1,336	4,566
		(%)^e^	(5.0)	(6.6)	(6.9)	(7.5)	(9.4)	(13.2)	(19.7)	(22.7)	(12.9)
	Meniscectomy & repair	Cases	6	7	4	14	23	25	33	51	163
		(%)^e^	(0.2)	(0.2)	(0.1)	(0.4)	(0.4)	(0.4)	(0.6)	(0.9)	(0.5)
	Total		2,968	2,942	2,805	3,385	5,537	6,035	5,717	5,881	35,270
60 ≤	Meniscectomy	Cases	1,656	1,800	1,870	2,390	3,892	4,198	3,998	4,022	23,826
		(%)^e^	(99.4)	(99.3)	(98.9)	(99.0)	(98.3)	(97.2)	(95.6)	(94.3)	(97.2)
	Meniscus repair	Cases	10	12	21	24	62	115	179	237	660
		(%)^e^	(0.6)	(0.7)	(1.1)	(1.0)	(1.6)	(2.7)	(4.3)	(5.6)	(2.7)
	Meniscectomy & repair	Cases	0	0	0	0	4	5	5	8	22
		(%)^e^	(0.0)	(0.0)	(0.0)	(0.0)	(0.1)	(0.1)	(0.1)	(0.2)	(0.1)
	Total		1,666	1,812	1,891	2,414	3,958	4,318	4,182	4,267	24,508

^a^ the Japanese academic year begins April 1 and ends March 31

^b^ July to December

^c^ July to March

^d^ April to March

^e^ proportion of all meniscus surgery during the data collection period.

**Table 3:** Annual trends in meniscectomy and meniscus repair categorized by injured compartment.

	Japanese academic year^a^		2007	2008	2009	2010	2011	2012	2013	2014	Total
Compartment^b^	Survey periods (months)		6^c^	6^c^	6^c^	9^d^	12^e^	12^e^	12^e^	12^e^	75
Medial	Meniscectomy	Cases	2,163	2,342	2,234	2,759	4,601	5,001	4,512	4,413	28,025
		(%)^f^	(95.0)	(93.6)	(94.8)	(92.8)	(91.8)	(87.8)	(82.7)	(81.0)	(88.4)
	Meniscus repair	Cases	109	158	122	207	391	677	925	1,014	3,603
		(%)^f^	(4.8)	(6.3)	(5.2)	(7.0)	(7.8)	(11.9)	(17.0)	(18.6)	(11.4)
	Meniscectomy & repair	Cases	4	1	1	6	19	15	18	20	84
		(%)^f^	(0.2)	(0.0)	(0.1)	(0.2)	(0.4)	(0.3)	(0.3)	(0.4)	(0.3)
	Total		2,276	2,501	2,357	2,972	5,011	5,693	5,455	5,447	31,712
Lateral	Meniscectomy	Cases	1,392	1,385	1,436	1,672	2,689	2,967	2,697	2,689	16,927
		(%)^f^	(92.1)	(90.8)	(89.9)	(87.3)	(85.1)	(79.6)	(72.7)	(69.9)	(80.6)
	Meniscus repair	Cases	117	139	161	237	458	741	983	1,116	3,952
		(%)^f^	(7.7)	(9.1)	(10.1)	(12.4)	(14.5)	(19.9)	(26.5)	(29.0)	(18.8)
	Meniscectomy & repair	Cases	3	2	1	7	14	18	31	44	120
		(%)^f^	(0.2)	(0.1)	(0.1)	(0.4)	(0.4)	(0.5)	(0.8)	(1.1)	(0.6)
	Total		1,512	1,526	1,598	1,916	3,161	3,726	3,711	3,849	20,999
Bicompartmental	Meniscectomy	Cases	20	15	26	33	48	68	53	56	319
		(%)^f^	(90.9)	(78.9)	(83.9)	(78.6)	(88.9)	(81.0)	(77.9)	(70.9)	(79.9)
	Meniscus repair	Cases	1	4	3	7	5	16	11	15	62
		(%)^f^	(4.5)	(21.1)	(9.7)	(16.7)	(9.3)	(19.0)	(16.2)	(19.0)	(15.5)
	Meniscectomy & repair	Cases	1	0	2	2	1	0	4	8	18
		(%)^f^	(4.5)	(0.0)	(6.5)	(4.8)	(1.9)	(0.0)	(5.9)	(10.1)	(4.5)
	Total		22	19	31	42	54	84	68	79	399

^a^ the Japanese academic year begins April 1 and ends March 31

^b^ patients with missing injury data were excluded from the analysis

^c^ July to December

^d^ July to March

^e^ April to March

^f^ proportion of all meniscus surgery during the data collection period.

The age of the entire cohort exhibited a bimodal distribution, with peaks at 10–19 years and 60–69 years (Table 4). Subgroup analysis revealed that the bimodal distribution was maintained in the meniscectomy group with peaks at the same ages, but there was a monomodal age distribution of patients undergoing meniscus repair that peaked at 10–19 years. For patients under the age of 49 years, the proportion of males undergoing meniscus surgery was greater; however, patients 50 years of age or older were more likely to be females. The site of injury also appeared to be influenced by age: medial tears were most common at 60–69 years of age and lateral tears at 10–19 years of age.

**Table 4:** Age distribution of patients undergoing meniscus surgery, categorized by procedure, injured compartment and sex.

	Age categories		< 10	10–19	20–29	30–39	40–49	50–59	60–69	70–79	80 ≤	Total
	Total	Cases	590	14,362	8,375	9,723	11,483	14,064	14,690	8,369	1,449	83,105
		(%)^a^	(0.7)	(17.3)	(10.1)	(11.7)	(13.8)	(16.9)	(17.7)	(10.1)	(1.7)	
Type of procedures	Meniscectomy	Cases	462	8,939	5,542	7,530	9,815	13,196	14,172	8,221	1,433	69,310
		(%)^a^	(0.7)	(12.9)	(8.0)	(10.9)	(14.2)	(19.0)	(20.4)	(11.9)	(2.1)	
	Meniscus repair	Cases	116	5,306	2,768	2,129	1,604	833	507	137	16	13,416
		(%)^a^	(0.9)	(39.5)	(20.6)	(15.9)	(12.0)	(6.2)	(3.8)	(1.0)	(0.1)	
	Meniscectomy & repair	Cases	12	117	65	64	64	35	11	11	0	379
		(%)^a^	(3.2)	(30.9)	(17.2)	(16.9)	(16.9)	(9.2)	(2.9)	(2.9)	(0.0)	
Injured compartments^b^	Medial	Cases	10	2,455	2,102	3,765	5,101	6,600	7,013	4,008	658	31,712
		(%)^a^	(0.0)	(7.7)	(6.6)	(11.9)	(16.1)	(20.8)	(22.1)	(12.6)	(2.1)	
	Lateral	Cases	449	6,835	2,974	2,501	2,429	2,507	2,122	1,017	165	20,999
		(%)^a^	(2.1)	(32.5)	(14.2)	(11.9)	(11.6)	(11.9)	(10.1)	(4.8)	(0.8)	
	Bicompartmental	Cases	0	44	25	32	47	86	95	58	12	399
		(%)^a^	(0.0)	(11.0)	(6.3)	(8.0)	(11.8)	(21.6)	(23.8)	(14.5)	(3.0)	
Sex	Male	Cases	277	7,801	5,810	6,507	6,558	6,454	5,887	2,992	538	42,824
		(%)^a^	(0.6)	(18.2)	(13.6)	(15.2)	(15.3)	(15.1)	(13.7)	(7.0)	(1.3)	
	Female	Cases	313	6,561	2,565	3,216	4,925	7,610	8,803	5,377	911	40,281
		(%)^a^	(0.8)	(16.3)	(6.4)	(8.0)	(12.2)	(18.9)	(21.9)	(13.3)	(2.3)	

^a^ proportion of the age category for all ages

^b^ patients with missing injury data were excluded from the analysis.

## Discussion

Between 2007 and 2014 in Japan, arthroscopic meniscus repair became increasingly popular at the expense of meniscectomy. In the last survey year, the proportion of patients under 30 years old undergoing meniscus repair exceeded that of patients undergoing meniscectomy. The age distribution of patients undergoing meniscectomy presented bimodal peaks at adolescence and at early old age, while meniscus repair showed a single peak in adolescence.

There have been several nationwide studies describing medium- to long-term trends in meniscus surgery. Investigators from Denmark examined the annual incidence of arthroscopic meniscus procedures from 2000 to 2011 using the Danish National Patient Register [33]. The incidence of meniscus surgery increased over time in all age groups, with the largest increase observed in patients over 55 years old, but surgical technique was not recorded; consequently, the proportion undergoing meniscectomy or meniscus repair could not be calculated. The second study examined the PearlDiver Patient Record Database, which represents approximately 9% of the population under 65 years of age in the United States, between 2004 and 2009 [32]. This cross-sectional analysis did not demonstrate any significant change in the incidence of lateral meniscus repair, or medial and lateral meniscectomy, although the incidence of medial meniscus repair significantly decreased during the study period. The third report also used the PearlDiver Patient Record Database, albeit more recently (2005 to 2011) [16]. The proportions of meniscus repair stayed at around 6% of all meniscus procedures in every survey year (e.g., 5.8% [3,197 of 54,900 cases] at 2005, 5.5% [3,428 of 62,395 cases] at 2008, and 6.2% [3,561 of 57,671 cases] at 2011); even among patients under 25 years of age, the proportion undergoing meniscus repair did not show a remarkable increasing trend (e.g., 23.4% [1,362 of 5,812 cases] at 2005, 24.3% [1,737 of 7,135 cases] at 2008, and 27.4% [1,967 of 7,183 cases] at 2011). The fourth and most recently published paper on this topic investigated the American Board of Orthopaedic Surgery database from 2004 to 2012 [31], and compared the trends of cases of meniscus repair and meniscectomy per surgeon in training; the rate of meniscus repair increased from 1.6 to 2.2 cases per surgeon, while that of meniscectomy decreased from 16.8 to 14.0 cases per surgeon. This study concluded that these findings supported the shift of practice patterns among arthroscopic surgeons toward meniscus preservation. However, the proportion of meniscus repair among meniscus procedures was still much lower than that of meniscectomy, and it is estimated that the result of this study did not reflect the nationwide trend because only the cases in teaching hospitals were included into the cohort.

We found the largest ever increase in the proportion of patients with meniscus injury undergoing meniscus repair, especially in young patients (< 30 years of age), which was a distinct trend compared to the previous studies above. This may reflect the growing awareness of the importance of meniscus preservation among arthroscopic surgeons in Japan. Meanwhile, a significant decrease in the proportion of patients undergoing meniscectomy was also observed, potentially explained by the growing body of evidence that meniscectomy does not improve the outcome of degenerative tears [24–29]. This is in contrast with two of the previous studies, which reported stable or increasing numbers of patients undergoing meniscectomy [16,32], and chimes with one of them [31].

Meniscus tears generally fall into one of two categories [16,23,37], degenerative tears (which become more common with aging) [38] and traumatic tears (which are often associated with sports injury and consequently occur in younger patients) [16,23]. In our cohort there was a bimodal age distribution, with surgery being undertaken most often in the teens and the sixties. All other previous reports have demonstrated a unimodal age distribution, with surgery most common in middle or older age [16,32,33]. Our finding reflects the relatively higher incidence of meniscus surgery in young patients and the lower incidence in middle-aged and older patients in Japan. This trend is strengthened by the most recent census [39]; those 60–64 years of age accounted for 7.9% of the total population, the largest of the all age categories, while those aged 15–19 years comprised only 4.8% of the total population.

Our finding that meniscus repair was most commonly carried out in patients aged 10–19 years is consistent with previous reports [16,32], but the bimodal age distribution for meniscectomy, with peaks at 10–19 and 60–69 years of age is not. Indeed previous studies have found that meniscectomy was undertaken most often in middle or older age [16,32]. This finding may be explained by the falling frequency of meniscectomy in middle-aged and older patients, and the higher prevalence of discoid meniscus in Japan [40,41], which is a major cause for meniscectomy in children and adolescents [40–42].

In the subgroup analyses, we found that medial tears occurred approximately 1.5 times more often than lateral ones, which is comparable to a 2:1 ratio reported by Campbell and colleagues [43]. Then, we found that medial tears were most common in those aged 60–69 years, while teenagers were more susceptible to lateral tears. These findings were consistent with but more pronounced than a nationwide study in the United States [32], and supported the hypothesis that medial tears are a degenerative injury, whereas lateral tears mainly arose from trauma or as a complication of a lateral discoid meniscus [42,44]. Further, this difference in age distribution between site of injury accounted for the larger proportions in repair of lateral meniscus than medial one.

Among patients younger than 50 years old in our cohort, males had a greater proportion of meniscus surgery than females. This trend is compatible with the previous study describing that young males may more frequently engage in high-risk activities that was susceptible to meniscus injuries [16]. By contrast, the majority of patients over the age of 50 years undergoing arthroscopic meniscus procedures were females. As degenerative meniscus tears are strongly associated with the initial stages of knee joint osteoarthritis [23], this finding corroborates that of the ROAD study [45], a population-based cohort study in Japan, which found that females were twice as likely as males to exhibit radiographic evidence of knee osteoarthritis.

Our study had several limitations. First, miscoding of procedures may have occurred, leading to flawed data. While this is a concern for any study using administrative data as verification of the data for each patient is not possible, we nonetheless assume that the rate of coding error in the DPC is low because data are entered by physicians and subject to internal audit. Second, meniscus procedures performed simultaneously with anterior cruciate ligament (ACL) reconstruction cannot normally be claimed together in the Japanese healthcare system as only the fees for ACL reconstruction can be reimbursed; consequently, these cases were excluded from our study cohort. This exclusion of meniscus procedures concomitant with ACL reconstruction might have some influence on the annual trends and the age distributions shown in the present study. However, as the majority of ACL injury occur in youth [16], this would not alter the increases in the proportions undergoing meniscus repair shown in Table 1 and Table 3, or the peaks at adolescence of the whole cohort, the meniscectomy group and the meniscus repair group shown in Table 4. Third, the injured compartment was not recorded in a substantial number of cases, likely because this is not compulsory for reimbursement in the DPC system. This would also affect the annual trends and the age distributions categorized by site of injury. Fourth, the DPC database is a patient discharge and administrative claims database, and lacks outpatient data. Therefore, outpatient arthroscopic surgery was excluded from our study. Although a large part of knee arthroscopy is done on an outpatient basis in some countries, such as the United States [46], almost all arthroscopic surgery is still performed in an inpatient setting in Japan. Fifth, the data collection periods for the DPC database were limited to the latter half of the year (i.e., July to December) from 2007 to 2010, while the data has been collected throughout the year since 2011. However, we preliminarily confirmed that there was little difference in the annual proportions of meniscectomy and meniscal repair between April to March and July to December; thus, we included all of the cases registered in the DPC database. Sixth, the DPC database does not provide detailed clinical information, such as activity level and operative technique. Finally, there may be some selection bias because not all of the hospitals in Japan adopt the DPC system; contribution to the database by academic hospitals is mandatory, whereas community hospitals participate only voluntarily.

## Conclusions

There were a rapid increase in the proportion of patients undergoing meniscus repair, and a significant fall in the proportion undergoing meniscectomy in Japan between 2007 and 2014, especially in younger patients, which were characteristic trends for the country. Meniscectomy was performed most frequently in early old age followed by adolescence, while meniscus repair was most often performed during adolescence.
